# Genome-wide transcriptome profiling of ex-vivo precision-cut slices from human pancreatic ductal adenocarcinoma

**DOI:** 10.1038/s41598-020-65911-3

**Published:** 2020-06-03

**Authors:** Mehran Ghaderi, Carlos Fernández Moro, Soledad Pouso Elduayen, Emilie Hultin, Caroline Sophie Verbeke, Mikael Björnstedt, Joakim Dillner

**Affiliations:** 1 Department of Laboratory Medicine, Division of Pathology F46, Karolinska Institutet, Karolinska University Hospital, Huddinge, SE-141 86 Stockholm Sweden; 2 Department of Clinical Pathology and Cytology, Karolinska University Hospital, Stockholm, SE-141 86 Sweden; 3 Institute of Clinical Medicine, University of Oslo, Oslo, NO-0316 Norway

**Keywords:** Cancer models, Transcriptomics

## Abstract

Ex-vivo tumor tissue culture systems are used as models to test specific anti-cancer drugs. Their main advantage is that they are closely comparable with the *in vivo* tumor in their host organism. We previously reported that precision-cut organotypic tissue slices of pancreatic ductal adenocarcinoma (PDAC) can be successfully cultured ex-vivo for at least 4 days. In order to study how culturing might affect transcription patterns, we now performed genome-wide transcriptome profiling of both baseline (0 h) and explanted tumors at daily intervals (24, 48 and 72 h) after start of culturing. The total-RNA from five samples of surgically resected human PDAC tumors at baseline and at different time points in culture was sequenced. Differential gene expression analysis of the whole transcriptome, testing 58,713 genes and over 206,000 transcripts, found that only a small number of genes showed significant changes in expression between baseline and cultured samples. The cultured tumor slices showed upregulation of a median of 12, 10 and 15 genes and downregulation of a median of 15, 12 and 25 genes at 24, 48 and 72 h in culture, respectively. One sample had morphologically increasing loss of tissue viability (range 0–18%). The vascular endothelial growth factor A (VEGFA) was significantly upregulated during the entire culture period in this case. Pathway over-representation analysis suggested that VEGFA together with the PTGS2 gene were upregulated at the same time as HIF-1-triggered cell apoptosis via NF-ĸB and the AP-1 activating factor was induced. Indeed, increased areas of apoptotic lesions were visible in this sample after 24 hours of culture. In conclusion, genome-wide transcriptome analysis supports that ex-vivo cultured tissue slices of PDAC may be a representative model of the original tumor. Transcriptome analysis was found to be a valuable complement to morphology for evaluation of ex-vivo cultures of PDAC.

## Introduction

Pancreatic cancer is currently the fourth leading cause of cancer-related death in the West and predicted to rank second by 2030^1^. Discrepancies in pancreatic cancer incidence between countries suggest that environmental and geographical factors may play a substantial role as risk factors for the disease^2^. Pancreatic cancer is usually diagnosed at advanced stages and characterized by aggressive tumor biology and pronounced resistance to treatment, which result in an overall 5-year survival rate of <7%^3^. PDAC tumors with distant metastases are generally not resected due to their dismal prognosis and surgical removal is indicated only for localized cancer. Risk factors are a family history of pancreatic cancer, smoking, obesity, chronic pancreatitis and genetic alterations, e.g. in BRCA1, BRCA2, PALB2 and ATM^4^.

Most pancreatic cancers have sporadic gene alterations, such as amplifications, deletions, translocations, inversions, frame-shifts, and substitutions. Mutations of multiple genes in pancreatic cancer occur in over 70% of all diagnosed cases. Most of the genetic alterations cause overexpression or activate signal transduction pathways^5^. As a result, translated oncoproteins or suppressor cellular regulatory mechanisms force cells into dysregulated growth^6–8^.

Histopathologically, most PDACs tumors are characterized by the presence of a prominent desmoplastic stroma with microenvironment that is composed of an admixture of acellular and cellular elements, including the extracellular matrix, mesenchymal cells (mainly cancer associated fibroblasts) and immune cells. The tumor microenvironment has pleiotropic tumor-promoting effects and contributes to an immune suppressive milieu, which can limit treatment efficacy^9,10^. Since the microenvironment in pancreatic cancer has unique features that directly impact the molecular signatures of the tumor cells, it is important to study the global molecular events that take place in the tumor tissues^11,12^.

Transcriptome sequencing uses massive parallel sequencing technologies to measure tissue RNA expression. Sequence data can be aligned to a reference genome to build full-length transcripts. Annotated transcripts are used to study differentially expressed genes while non-mapped reads can be analyzed to study fusion genes or viral transcripts. Transcriptome sequencing data can also be used to define novel transcripts leading to discovery of neoantigens^13–15^.

Earlier, Misra and Moro *et al*., showed that pancreatic cancer cells, the tumor microenvironment and the interspersed remnants of nonneoplastic pancreas in 350 µm thick slices maintained their structural integrity, phenotypic characteristics and functional activity when kept in culture for at least 4 days^16^.

In the present study, we employed deep sequencing to characterize the transcriptome of five cases of PDAC that were selected based on the different degree of cell death in the tissues and tumor grade. We used freely available, open source bioinformatic tools to perform differential gene expression and pathway enrichment analysis. We hypothesized that tissue viability (and its loss) in ex-vivo cultured tissue slices would be reflected in the differences in transcription of genes involved in pathways related to cell death.

## Results

We sequenced FFPE tissue from five tumor samples at baseline and their matched cultured tissue slices at 24, 48, and 72 h. In total 5 baseline tumor samples and 15 corresponding organotypic (OT) cultures were assessed.

**Figure 1:**
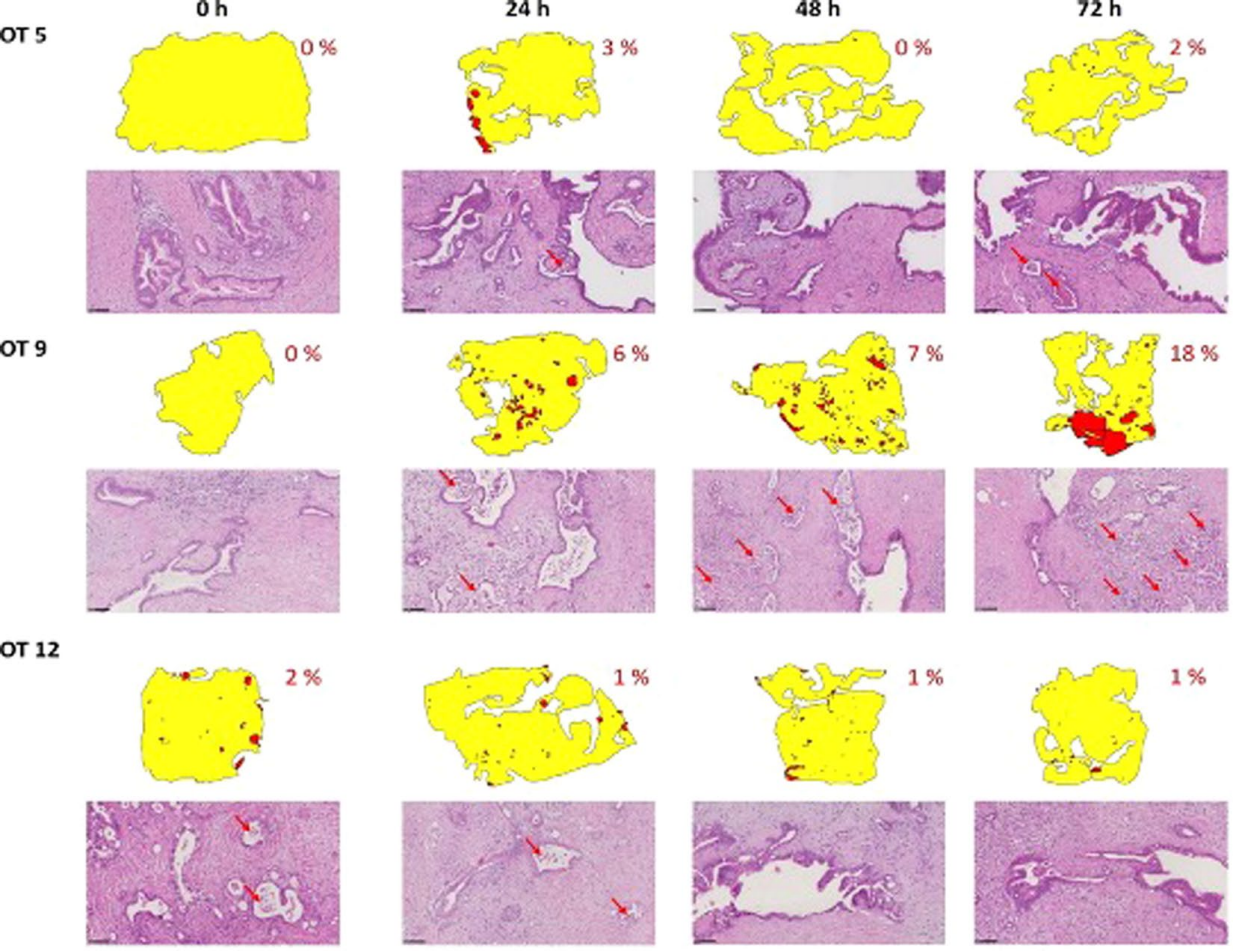
Morphology-based analysis of tissue viability in the baseline (0 h) and cultured (24–2 h) tissue slices of PDAC samples OT5, OT9 and OT12. Non-viable tissue regions are indicated as red areas in the slice diagrams, their total percentages (with respect to the whole slice area) in red font and by red arrows in representative photomicrographs (hematoxylin-eosin staining, bar line: 100 μm).

Two OT cultures were from well differentiated and three from moderately to poorly differentiated ductal adenocarcinomas. These were selected from the previously published series (Misra and Moro *et al*.) for being histomorphologically comparable (same tumor type and grouped by grade of differentiation) but showing different levels of tissue viability during culture^16^. In particular, four cultures (OT1, OT5, OT11 and OT12) showed no or minimal loss of viability (range 0–6%), while one (OT9) showed mild to moderate loss of tissue viability (range 0–18%) (Fig. 1. legend provided). The tumor cell outgrowth along the edge of the tissue slices ranged from 71–100% (of the perimeter) for the four cultures with high tissue viability and 55–76% for the one with viability loss. Patient gender and clinicopathological data as well as the percentage ranges of non-viable tissue for each OT culture are presented in Table 1.

**Table 1:** Clinicopathological data and histological features of the slice cultures.

Culture ID	Age	Gender	Grade of tumor differentiation	*Non-viable tissue (range %)	**Tumor cell outgrowth (range %)
OT1	68	Male	Moderate	0–3	7–15
OT5	74	Male	Well	0–3	71–100
OT9	62	Male	Well	0–18	55–76
OT11	47	Female	Moderate to poor	0–6	23–47
OT12	80	Male	Moderate	1–2	79–100

*Range % of non-viable tissue with respect to the entire tissue slice area, from time points 0 h to 72 h.

**Range % of tumor cell outgrowth with respect to the tissue slice perimeter, from time points 24 h to 72 h.

Based on human assembly Homo sapiens GRCh38.95 annotations, 58,713 genes (coding and non-coding) and 206,487 gene transcripts were tested. Transcript isoform test detected no statistically significantly differentially expressed isoforms at any time point.

To analyze the differences in gene expression, each FFPE block prepared at time points 24 h, 48 h, and 72 h was compared to the baseline FFPE tumor tissue sample. Using TopHat2/Cuffdiff a limited number of differentially expressed genes were selected when allowing a false discovery rate of 0.05 (Table 2).

**Table 2:** Total number of differentially expressed genes (p-value 0.05) in examined OT samples after ex-vivo harvest time from 24 to 72 hours.

Sample	24 h	48 h	72 h
OT1	19 (7$\downarrow$12$\uparrow$)	20 (7$\downarrow$13$\uparrow$)	21 (6$\downarrow$15$\uparrow$)
OT5	36 (15$\downarrow$21$\uparrow$)	32 (26$\downarrow$6$\uparrow$)	47 (26$\downarrow$21$\uparrow$)
OT9	52 (15$\downarrow$37$\uparrow$)	41 (10$\downarrow$31$\uparrow$)	56 (38$\downarrow$18$\uparrow$)
OT11	0 (0$\downarrow$0$\uparrow$)	33 (23$\downarrow$10$\uparrow$)	40 (25$\downarrow$15$\uparrow$)
OT12	23 (14$\downarrow$9$\uparrow$)	20 (12$\downarrow$8$\uparrow$)	15 (11$\downarrow$4$\uparrow$)

$\uparrow$indicating upregulation and $\downarrow$indicating downregulation of genes in compare to the baseline tumor.

### Over-representation pathway analysis

To evaluate if any of the differentially expressed genes (DEGs) were associated significantly with certain pathways, over-representation analysis was performed. Those genes that were expected to be in at least in one pathway are presented in Table 3 with symbols to show if they were up- or downregulated. Overall results from the top 3 significantly estimated pathways for all samples are presented in Table 4. For sample OT11 no differentially expressed genes were detected after 24 h of culture. Accordingly, none of the mapped entities (18 out of 20) after 48 h of culture for sample OT12 were over-represented in an enriched pathway.

**Table 3:** Alphabetically ordered and white-spaced list of OT-sample transcripts (HGNC gene symbol used) that were over-represented in at least in one enriched pathway ConsensuspathDB-set.

Sample	24 h	48 h	72 h
OT1:	CHST2$\uparrow$ CRP$\uparrow$ CTSL$\uparrow$ CXCL8$\uparrow$ HES7$\uparrow$ HYDIN$\downarrow$ ITIH3$\uparrow$ MAPK10$\downarrow$ MUC5AC$\downarrow$ NDRG2$\downarrow$ RFX8$\uparrow$ RNA5S9$\uparrow$RUNX1T1$\downarrow$ SCD$\uparrow$ STC1$\uparrow$ VIPR1-AS1$\downarrow$ WARS$\uparrow$	ADRA2C$\uparrow$ AKR1C1$\uparrow$ CCL18$\uparrow$ CHCHD4$\uparrow$ CRP$\uparrow$ CTSL$\uparrow$ CYP3A5$\downarrow$ MMP14$\uparrow$ MUC5AC$\downarrow$ OCEL1$\uparrow$ OLFM4$\uparrow$ REG4$\downarrow$ SCD$\uparrow$ SERPINA1$\uparrow$ TEX41$\downarrow$ TMEM126A$\uparrow$ TRDN-AS1$\downarrow$ TXNIP$\downarrow$	ADRA2C$\uparrow$ CCL18$\uparrow$ CH25H$\uparrow$ CLGN$\uparrow$ CRP$\uparrow$ CTSL$\uparrow$ HYOU1$\uparrow$ MMP14$\uparrow$ MUC5AC$\downarrow$ OLFM4$\uparrow$ REG4$\downarrow$ SCD$\uparrow$ SERPINB4$\uparrow$ TNNT2$\downarrow$ TXNIP$\downarrow$ VIPR1-AS1$\downarrow$ WARS$\downarrow$ ZBBX$\downarrow$
OT5:	ABCC8$\downarrow$ ADM$\uparrow$ ADRA2C$\uparrow$ ALDH1A2$\uparrow$ CPA1$\downarrow$ CTSL$\uparrow$ GAL$\uparrow$ HMGCS2$\downarrow$ ICAM5$\uparrow$ IER3$\uparrow$ NDRG1$\uparrow$ NDRG2$\downarrow$ NOTUM$\uparrow$ PLIN2$\uparrow$ PNLIPRP1$\downarrow$ PPP1R15A$\uparrow$ SFRP1$\downarrow$ TTR$\downarrow$	ABCC8$\downarrow$ CPA1$\downarrow$ CREB3L3$\uparrow$ DLG2$\downarrow$ IGHA2$\downarrow$ IGHM$\downarrow$ IGKC$\downarrow$ INHBB$\downarrow$ MUC6$\downarrow$ NR4A1$\downarrow$ PNLIPRP1$\downarrow$ PPP1R1B$\downarrow$ SCN7A$\downarrow$ SLC30A8$\downarrow$ TCF4$\downarrow$ THBS1$\downarrow$	ABCC8$\downarrow$ ADM$\downarrow$ ADRA2C$\uparrow$ ALB$\uparrow$ CAPN13$\downarrow$ CREB3L3$\uparrow$ CTSL$\uparrow$ CXCL5$\downarrow$ DIAPH3$\downarrow$ FCGR3B$\downarrow$ FOLR2$\downarrow$ GAL$\uparrow$ ICAM5$\uparrow$ IGHM$\downarrow$ ITIH3$\downarrow$ JCHAIN$\downarrow$ MMP11$\downarrow$ PAX6$\downarrow$ PLIN2$\uparrow$ PNLIPRP1$\downarrow$ SLC28A2$\downarrow$ SPARCL1$\downarrow$
OT9:	ARC$\uparrow$ CTSL$\uparrow$ DUSP5$\uparrow$ F13A1$\downarrow$ FST$\downarrow$ FZD10$\uparrow$ GJA1$\uparrow$ GRIA1$\downarrow$ HK2$\uparrow$ IGFBP1$\uparrow$ LIF$\uparrow$ NOG$\uparrow$ OSM$\uparrow$ PLIN2$\uparrow$ PMAIP1$\uparrow$ PPP1R15A$\uparrow$ PTGS2$\uparrow$ TBX3$\uparrow$ TGFBI$\uparrow$ VEGFA$\uparrow$	ANK3$\downarrow$ CTSL$\uparrow$ FST$\uparrow$ GJA1$\uparrow$ IER3$\uparrow$ IL33$\uparrow$ PLIN2$\uparrow$ PMAIP1$\uparrow$ PTGS2$\uparrow$ TGFBI$\uparrow$ TXNIP$\downarrow$ UGT2A1$\downarrow$ VEGFA$\uparrow$	BRCA1$\downarrow$ CD44$\uparrow$ CFB$\uparrow$ CNKSR2$\downarrow$ ELN$\downarrow$ ENO1$\uparrow$ F13A1$\downarrow$ ITGA7$\downarrow$ MEF2C$\downarrow$ MFAP5$\downarrow$ MT-CO1$\downarrow$ MT-CO2$\downarrow$ MT-CYB$\downarrow$ MT-ND1$\downarrow$ MT-ND2$\downarrow$ MT-ND3$\downarrow$ MT-ND4$\downarrow$ MT-ND5$\downarrow$ MT-ND6$\downarrow$ MT-TC$\downarrow$ MT-TL1$\downarrow$ MT-TM$\downarrow$ MT-TQ$\downarrow$ MT-TV$\downarrow$ ORM1$\uparrow$ PAX6$\uparrow$ PNPLA7$\downarrow$ RPS4Y1$\downarrow$ ST3GAL6$\uparrow$ VEGFA$\uparrow$
OT11:	No differentially expressed genes were found after 24 hours of culture.	A2M$\downarrow$ ADRA2C$\uparrow$ AMY2A$\downarrow$ CCL21$\downarrow$ CPA1$\downarrow$ CPA2$\downarrow$ CRISP3$\downarrow$ CTGF$\downarrow$ CTSL$\uparrow$ CXCR4$\downarrow$ DUSP1$\downarrow$ EGR1$\downarrow$ FOS$\downarrow$ FOXA3$\uparrow$ HAGHL$\uparrow$ HLA-DQB2$\downarrow$ HYOU1$\uparrow$ IGHA1$\downarrow$ IGHA2$\downarrow$ IGHD$\downarrow$ IGKC$\downarrow$ JCHAIN$\downarrow$ MUC6$\downarrow$ PLAU$\uparrow$ PNLIP$\downarrow$ SCD$\uparrow$SERPINA1$\uparrow$SPARCL1$\downarrow$ STC1$\uparrow$ TRDN-AS1$\uparrow$ TSC22D3$\downarrow$ ZFP36$\downarrow$ ZNF331$\downarrow$	A2M$\downarrow$ ADRA2C$\uparrow$ CCL21$\downarrow$ CP$\uparrow$ CRP$\uparrow$ CTGF$\downarrow$ CX3CR1$\downarrow$ EGR1$\downarrow$ F5$\downarrow$ FOLR2$\downarrow$FOS$\downarrow$ IGFBP1$\uparrow$ IGHA1$\downarrow$ IGHA2$\downarrow$ IGHD$\downarrow$ IGHG1$\downarrow$ IGKC$\downarrow$ IGLC1$\downarrow$ JCHAIN$\downarrow$ MUC6$\downarrow$ NMU$\uparrow$ RGS1$\downarrow$ SCD$\uparrow$ SERPINA1$\uparrow$ SERPINB3$\uparrow$ SERPINB4$\uparrow$ SPARCL1$\downarrow$ TSC22D3$\downarrow$ ZFP36$\downarrow$
OT12:	COL21A1$\downarrow$ COL7A1$\uparrow$ CTSL$\uparrow$ F3$\uparrow$ FOLR2$\downarrow$ GALNT5$\uparrow$ LAMC2$\uparrow$ MAPK10$\downarrow$ PNLIPRP1 PPY$\downarrow$ THSD7B$\downarrow$ VIP$\uparrow$	None of the mapped entities (18 out of 20) were significantly over-represented in any pathway	CTSL$\uparrow$ MMP14$\uparrow$ TXNIP$\downarrow$

**Table 4:** Probability of the top 3 significantly suggested pathways according to the hypergeometric test in different databases.

		p-value	Pathway	Database	Mapped genes
OT1	24 h	0.000122	IL-17 signaling pathway - Homo sapiens (human)	KEGG	MAPK10; MUC5AC; CXCL8
	24 h	0.000182	Overview of nanoparticle effects	Wikipathways	CXCL8; CRP
	24 h	0.000431	nfkb activation by nontypeable hemophilus influenzae	BioCarta	CXCL8; MUC5AC
	48 h	0.000724	Collagen degradation	Reactome	MMP14; CTSL
	48 h	0.000784	Innate Immune System	Reactome	CRP; CTSL; TXNIP; OLFM4; SERPINA1; MUC5AC
	48 h	0.00154	Xenobiotics metabolism	EHMN	CYP3A5; AKR1C1
	72 h	0.000724	Collagen degradation	Reactome	MMP14; CTSL
	72 h	0.00392	Phenytoin (Antiarrhythmic) Action Pathway	SMPDB	HYOU1; TNNT2
	72 h	0.00548	Innate Immune System	Reactome	CRP; CTSL; TXNIP; OLFM4; MUC5AC
OT5	24 h	1.12e-05	Validated targets of C-MYC transcriptional repression	PID	HMGCS2; NDRG1; NDRG2; SFRP1
	24 h	0.000266	HIF-1-alpha transcription factor network	PID	ADM; PLIN2; NDRG1
	24 h	0.000877	Sympathetic Nerve Pathway (Neuroeffector Junction)	PharmGKB	ADM; ADRA2C
	48 h	1.92e-05	Inflammatory Response Pathway	Wikipathways	IGHA2; THBS1; IGHM
	48 h	0.0028	Cocaine addiction - Homo sapiens (human)	KEGG	PPP1R1B; CREB3L3
	48 h	0.00326	Peptide hormone metabolism	Reactome	INHBB; SLC30A8
	72 h	0.00153	Sympathetic Nerve Pathway (Neuroeffector Junction)	PharmGKB	ADM; ADRA2C
	72 h	0.00235	Degradation of the extracellular matrix	Reactome	MMP11; CAPN13; CTSL
	72 h	0.0026	Plasma lipoprotein remodeling	Reactome	CREB3L3; ALB
OT9	24 h	3.07e-06	Photodynamic therapy-induced HIF-1 survival signaling	Wikipathways	PTGS2; IGFBP1; PMAIP1; VEGFA
	24 h	7.54e-06	Interleukin-4 and Interleukin-13 signaling	Wikipathways	PTGS2; OSM; VEGFA; F13A1; LIF
	24 h	3.54e-05	HIF-1-alpha transcription factor network	PID	PLIN2; IGFBP1; HK2; VEGFA
	48 h	4.31e-05	Photodynamic therapy-induced HIF-1 survival signaling	Wikipathways	PTGS2; PMAIP1; VEGFA
	48 h	0.000368	Quercetin and Nf-kB- AP-1 Induced Cell Apoptosis	Wikipathways	PTGS2; VEGFA
	48 h	0.000661	S1P1 pathway	PID	PTGS2; VEGFA
	72 h	2.37e-11	Respiratory electron transport	Reactome	MT-CO2; MT-CO1; MT-CYB; …
	72 h	3.11e-11	Electron Transport Chain (OXPHOS system in mitochondria)	Wikipathways	MT-ND6; MT-ND5; MT-ND4;…
	72 h	1.56e-10	Respiratory electron transport, ATP synthesis by chemiosmotic coupling	Reactome	MT-CO2; MT-CO1; MT-CYB; …
OT11	48 h	2.48e-05	AP-1 transcription factor network	PID	FOS; DUSP1; EGR1; PLAU
	48 h	8.59e-05	Pancreatic secretion - Homo sapiens (human)	KEGG	AMY2A; PNLIP; CPA1; CPA2
	48 h	0.000126	ErbB1 downstream signaling	PID	FOS; ZFP36; EGR1; DUSP1
	72 h	1.33e-05	Post-translational protein phosphorylation	Reactome	SPARCL1; CP; F5; SERPINA1; IGFBP1
	72 h	2.69e-05	Regulation of Insulin-like Growth Factor (IGF) transport and uptake by Insulin-like Growth Factor Binding Proteins (IGFBPs)	Reactome	SPARCL1; CP; F5; SERPINA1; IGFBP1
	72 h	0.000316	IL6-mediated signaling events	PID	CRP; FOS; A2M
OT12	24 h	4.01e-06	Collagen formation	Reactome	LAMC2; COL7A1; CTSL; COL21A1
	24 h	2.56e-05	Assembly of collagen fibrils and other multimeric structures	Reactome	LAMC2; CTSL; COL7A1
	24 h	2.97e-05	Anchoring fibril formation	Reactome	COL7A1; LAMC2
	72 h	0.000147	Collagen degradation	Reactome	MMP14; CTSL
	72 h	0.00132	Degradation of the extracellular matrix	Reactome	MMP14; CTSL
	72 h	0.0065	VEGFA-VEGFR2 Signaling Pathway	Wikipathways	MMP14; TXNIP

Over-representation analysis for sample OT9, indicated that the gene sets PTGS2; IGFBP1; PMAIP1; and VEGFA could activate the therapy-induced HIF-1 survival signaling pathway followed by Quercetin, Nf-kB and AP-1 Induced Cell Apoptosis (48 h), the latter being in line with the histological detection of cell death/apoptosis (Fig. 1).

After 72 h, several mitochondrial transcripts were predominantly downregulated, suggesting a drop in oxidative phosphorylation, NADH dehydrogenase activity, and the respiratory electron transport system (Table 3).

The Cathepsin L gene transcript, CTSL was frequently upregulated in all cases. CTSL together with isoforms of MMP metalloproteinase and/or collagen formation factors were mapped in the Reactome database, mainly in pathways controlling the skeleton of the solid tissues (Tables 3 and 4).

### The long noncoding RNAs MALAT1 and NEAT1

High levels of transcripts of long non-coding lncRNAs, MALAT1 and NEAT1 (located on the long arm of chromosome 11) have been described in various cancer types including pancreatic cancer^17,18^. We found that both transcripts were highly expressed (based on FPKM values) in all examined samples (data not shown) but there were no overall expression changes of these transcripts during the incubations.

### Analysis of biologically relevant fusion transcripts

No possible oncogenic fusions were detected in sample OT5 at any time-point.

FusionCatcher detected “likely” fusion of CDKL5-NEAT1 in specimen OT9 after 48 h but this fusion did not contain sequences from exonic regions of the involved genes.

## Materials and methods

### Preparation of precision-cut tissue slices

This study was approved by the Regional Ethical Review Board, Stockholm (IRB diary number 2012/1657-31/4). In brief, as previously described (Misra and Moro *et al*.), fresh samples of pancreatic cancer tissue were cut into 350 µm thick slices using a vibrating blade microtome. The slicing process yielded between 12 and 17 slices per tissue sample. The first slice (baseline tissue slice, time point 0 h) was immediately fixed in formalin and embedded in paraffin. This was used as baseline for morphological (tissue viability, grade of tumor differentiation) assessments in the cultured tissue slices. The subsequent eight slices were placed on inserts and cultured at 37 °C in a humidified incubator in 41% O_2_ for up to 96 h. Every 24 h, duplicate tissue slices were harvested, formalin fixed and embedded in paraffin. The tissue slices were subsequently sectioned and processed for histology. Loss of tissue viability was determined on hematoxylin-eosin stained sections according to the percentage of necrotic and apoptotic areas with respect to the entire surface of the tissue slice. Among the duplicate slices for 24, 48 and 72 h, the one with higher tumor tissue content was selected for transcriptome analysis.

### Isolation of total-RNA and library preparation

RNA was extracted and treated with DNase from the formalin-fixed paraffin embedded (FFPE) 2×10 µm curls using the Maxwell RSC FFPE RNA kit (Promega, Madison, USA). Extracted RNA was quantified by Qubit 4.0 and RNA HS Assay Kit (Thermofisher Scientific, Waltham, USA). A maximum of fifty nanogram of extracted RNA was used to prepare cDNA libraries skipping the RNA fragmentation step. Whole transcriptome sequencing libraries were prepared using the Takara Smarter total-RNA Seq kit V2 Pico Input Mammalian (Takara Bio Inc, Kusato, Japan). Briefly, cDNA was prepared by random hexamer priming while preserving strand of origin information. During a first PCR amplification, full-length Illumina adapters, including barcodes were added. The ribosomal cDNA sequences (originating from rRNA) were cleaved in the presence of RNAse H and the mammalian-specific R-Probes. The remaining fragments were enriched via a second round of PCR amplification using primers universal to all libraries. The final library contained sequences allowing clustering on any Illumina flow cell. All libraries including a non-template control were quantified on Bioanalyzer (Agilent Technologies, Santa Clara, USA). To equalize sequencing input amount, each library was quantified by Qubit 4.0 HS Assay Kit (Thermofisher Scientific, Waltham, USA).

### NextSeq 500 sequencing, bioinformatic analysis and statistical evaluation

In total twenty normalized libraries from five PDAC were sequenced on a NextSeq. 500 Illumina system (Illumina, San Diego, USA). Paired-end cycle sequencing 2 × 75 was run on High Output V2 Kit which in total generated a median of 40 million raw paired-end reads/sample. Indices, included in the Illumina adapters, were used to demultiplex and assign raw sequence reads. Datasets were analyzed using the Chipster virtual bioinformatic interface at CSC Finland^19^ to process and analyze RNA data for gene expression. All sequences were quality-checked by FASTQC^20^, adapters were preprocessed and trimmed if necessary. To estimate the stability of the sequencing reactions insertion/deletion rate values were observed after FASTQ alignment by STAR using Homo sapiens genome version GRCh38.95 as ref. ^21^ (data not shown).

For comparative analysis of gene expression, TopHat2 alignment tool for paired-end reads followed by the Cufflinks algorithm package version 2.1.1 for assembly and Cuffdiff for differential expression^22^. Multiple GTF files were merged using Cuffmerge and sorted by chromosome and start position.

GTF and the corresponding BAM files were compared to the reference baseline sample using the Cuffdiff tool to study the DEGS of the potentially novel transfrags where at least one splice junction was shared with a reference transcript. To calculate the DEGS, Cuffdiff differences in FPKM (Fragment Per Kilobase Million) values were considered significant when p-values were equal or less than 0.05.

### Hypergeometric test for ConsensusPathDB

To predict the possible molecular functional and gene-pathway interactions, Hypergeometric distribution probability test (over-representation analysis) was used. This pathway analysis is described by the ConsensusPathDB (CPDB) created by Herwig *et al*.^23^. This test searches gene lists for over-represented pathways in the CPDB database at: http://cpdb.molgen.mpg.de/. The p-values of each pathway test reflect the significance of the observed overlap between the input gene list and the module’s members as compared to random expectations. CPDB is an open source database provided by the Max Planck Institute for Molecular Genetics and contains pathway information from 32 publicly available databases, including BioCarta, HumanCyc, Reactome, KEGG and WikiPathways.

### Detection of fusion genes and viral/phage transcripts

To analyze the RNA sequencing data for detection of somatic fusion transcripts, FusionCatcher_v1.10 (https://github.com/ndaniel/fusioncatcher), using human genome database v95 was installed and used on a local Linux server^24^. The command line option was used to enable FusionCatcher to search for fusions in raw paired-end FASTQ files, skipping the alignment tool BLAT and adding -V to the command line to engage and store unaligned reads to be able to BLAST for possible viral transcripts. Only fusion transcripts not labeled as “banned” in the output file (because of their known presence in healthy individuals) or involving intronic sequences and not associated with a protein product were considered as a novel finding.

### Ethics declaration

This study was approved by the Regional Ethical Review Board, Stockholm (IRB diary number 2012/1657-31/4). Written informed consent was obtained from all patients prior to surgery. All study methods were performed in accordance with the relevant guidelines and regulations.

## Discussion

Sequencing of the total transcriptome from surgically resected human PDAC at four time points in culture in vitro revealed that limited number of genes (median 10 to 25) exhibited altered expression after ex-vivo culture. Among those, the pancreas-specific transcripts (PNLIPRP1: pancreatic lipase related protein 1 and CPA1: carboxypeptidase A1 and CPA2) were suppressed.

In this study modern deep sequencing with short reads was used. In this way a very deep transcriptional mapping could be performed, even in specimens that may contain fragmented ribonucleic acids. The fact that we found similar expression levels in the explanted tumor for >200,000 transcripts indicates that the analysis was both deep and comprehensive.

Tumor dissection to enrich for certain cell types was not performed, hence it was not possible to study the contribution or representation of the cellular compartment expression in diverse pathways. Rather, we studied the overall expression from all cellular components within the tissue including the tumor, stroma, infiltrating immune cells and nonneoplastic pancreatic tissue. In this way, it was possible to capture a genome-wide image of globally expressed genes, reflecting the molecular events that take place in the tumor tissue and its surrounding microenvironment.

Davies EJ. *et al*. also studied precision-cut slices prepared from human and animal models of different cancer types^25^. They investigated tissue viability and proliferation at different oxygen levels and analyzed the expression of a large set of RNA molecules that are involved in different cellular mechanisms, such as apoptosis, DNA damage and repair. They concluded that ischaemia during transportation from the surgical theatre and mechanical slicing (baseline tissue slice, timepoint 0 h) had little impact on stress gene expression, but that cultivation induced changes in gene and protein expression. They observed that atmospheric oxygen concentration is suitable for tissue slice culture and that the slice thickness has no impact on O_2_ diffusion and its availability to the tissue. Consistent with that, a previous study from our group showed no significant differences in tissue viability, proliferation, or metabolic activity between matched tissue slices cultured in atmospheric (21% O2) or hyperoxic (41% O2) conditions^16^. Davies EJ. *et al*. also showed overexpression of HIF-1α by immunohistochemistry in association with hypoxia and stress of the tissue slice compartments. In line with this observation, VEGFA and the HIF-1α signaling network were upregulated in the cultured slices of sample OT9 that exhibited increased amounts of apoptotic cell death. The vascular growth factor A, encoded by the *VEGFA* gene, is a key factor in angiogenesis and increased vascular permeability^26^. It can be speculated that upregulation of VEGFA was induced in hypoxic conditions because “HIF-1-induced” cell apoptosis via NF-ĸB and the AP-1 activating factor was suggested as a possible pathway.

Altogether, these findings support the role and value of assessing HIF-1α activity for the detection of cellular stress in the cultured tissue slices.

In the current study, the analysis of the total cellular transcriptome assessed the impact of the new environment on the cultured pancreatic cancer tissue. Ex-vivo model systems are influenced largely by culture conditions such as varying oxygen tension and absence of vital cellular stimulatory cues.

Future studies linking transcriptome data and morphological phenotypes of cancer tissue could be of value to shed light on the molecular origins of intratumoral heterogeneity.

## Conclusions

Genome-wide transcriptome sequencing revealed transcriptional changes only in a limited number of genes during tissue culture ex vivo. Changes were mainly seen in genes involved in biological processes whose molecular function can affect the stabilization or upregulation of cellular compartments. Consistent with histomorphological analysis, upregulation of pathways related to cell death/apoptosis were observed in a cultured tumor with decreased tissue viability, while they were not upregulated in the tumor cultures with minimal tissue loss.

The present analysis supports the transcriptional stability of the tissue slices during ex-vivo culture and its ability to reflect changes related to cell death. This is of special relevance in order to successfully address and correctly assess biological processes and drug responses in slice cultures. In addition, these findings support the use of transcriptomic analysis as a valuable tool for evaluation of ex-vivo cultures of PDAC.
